# End growth results analysis related to Risser score, Cobb degrees, and curve types at the beginning of the treatment

**DOI:** 10.1186/1748-7161-8-S1-O10

**Published:** 2013-06-03

**Authors:** S Donzelli, F Zaina, S Negrini

**Affiliations:** 1 ISICO (Italian Scientific Spine Institute), Milan, Italy; 2 University of Brescia, Brescia, Italy; 3 IRCCS Don Gnocchi, Milan, Italy

## Background

Scoliosis treatment is the science of prediction and estimation.The strategy is based on the magnitude,and pattern of the deformity, both related to age and Risser Score, to predict the potential progression. The efficacy of the conservative treatment of scoliosis is known, and analysis of factors potentially influencing the results is surely interesting.

## Aim

To assess the final results stratified according to curve magnitude, Risser score, curves type, gender and age, of a prospective set of patients treated in a centre fully dedicated to the conservative treatment of Adolescent Idiopathic Scoliosis AIS.

## Method

Study Design: Retrospective study. Population. 388 (31 males) patients respecting these inclusion criteria: AIS diagnosis, Risser test 0-3; all Cobb degrees; no prior treatment; who had reached the end of treatment since our institute database start in 2003. Methods: Clinical and radiographic (Cobb degrees) data at the beginning of treatment have been compared to end growth results. Treatments: All patients were treated respecting SOSORT standard of conservative treatment with observation, exercises, soft and rigid braces.

## Results

At the start of treatment worst curves corresponded to highest Risser test. With treatment, the percentage of unchanged patients remained almost stable (40-50%), while progression was higher in Risser 0 then at other Risser stages (16.6% vs 7.4-9.9%); highest rates of improvement appeared at Risser 1 or 3 (45-47% vs 33-39%). Patients who begin the therapy at Risser 0 have a higher probability to end treatment under 30° Cobb. The probability of curve progression is highest at the lowest and highest initial sizes of curve (i.e. below 20° or over 40°).In this sample of treated patients, the rate of progressed curves was very low, with a high rate of stabilized and improved curves. The total number of patients who finished with curves over 30°C increases proportionally with a starting Risser Score. Considering curve's type, age and gender we didn't find differences in final results.

## Conclusion

The efficacy of conservative treatment has been demonstrated in some previous studies. Our data confirms this aspect, with a trend of all final results (82.6% below 30°C), telling us that therapy is the most important predictive factor. As expected, the lower the age and Risser at start the lower the curve magnitude, and the best the final results, confirming the importance of early AIS detection.
